# IKK/NF-κB signaling contributes to glioblastoma stem cell maintenance

**DOI:** 10.18632/oncotarget.12507

**Published:** 2016-10-06

**Authors:** Amanda L. Rinkenbaugh, Patricia C. Cogswell, Barbara Calamini, Denise E. Dunn, Anders I. Persson, William A. Weiss, Donald C. Lo, Albert S. Baldwin

**Affiliations:** ^1^ Department of Pathology and Laboratory Medicine, University of North Carolina, Chapel Hill, NC, USA; ^2^ Lineberger Comprehensive Cancer Center, University of North Carolina, Chapel Hill, NC, USA; ^3^ Chordoma Foundation, Durham, NC, USA; ^4^ Center for Drug Discovery and Department of Neurobiology, Duke University Medical Center, Durham, NC, USA; ^5^ Helen Diller Family Comprehensive Cancer Center and Department of Neurology, University of California, San Francisco, CA, USA; ^6^ Department of Neurological Surgery and Brain Tumor Research Center, University of California, San Francisco, CA, USA

**Keywords:** NF-κB, glioblastoma, cancer stem cells, tumor-initiating cells

## Abstract

Glioblastoma multiforme (GBM) carries a poor prognosis and continues to lack effective treatments. Glioblastoma stem cells (GSCs) drive tumor formation, invasion, and drug resistance and, as such, are the focus of studies to identify new therapies for disease control. Here, we identify the involvement of IKK and NF-κB signaling in the maintenance of GSCs. Inhibition of this pathway impairs self-renewal as analyzed in tumorsphere formation and GBM expansion as analyzed in brain slice culture. Interestingly, both the canonical and non-canonical branches of the NF-κB pathway are shown to contribute to this phenotype. One source of NF-κB activation in GBM involves the TGF-β/TAK1 signaling axis. Together, our results demonstrate a role for the NF-κB pathway in GSCs and provide a mechanistic basis for its potential as a therapeutic target in glioblastoma.

## INTRODUCTION

Glioblastoma multiforme (GBM) is the most common primary brain tumor in adults, but despite multimodal treatment combining surgery, radiation, and chemotherapy, the median survival for patients remains under 15 months [1]. Pathologically, GBM is described as a heterogeneous tumor type with high levels of angiogenesis and invasion [2]. Recent studies provide evidence for a population of cancer stem cells (CSCs) within GBM contributing to this heterogeneity [3, 4]. These cells (hereafter called GSCs) carry neural stem cell markers such as CD133, self-renew over serial passages, and differentiate into multiple lineages. As with tumor-initiating cells described in other cancers [5–10], GSCs have been shown to be invasive and resistant to both radiation and chemotherapy [11–14]. As such, there is significant interest in understanding the biology of the GSC population to identify potential novel therapeutic targets to improve disease control.

NF-κB is a family of transcription factors consisting of five members: p65 (RelA), RelB, c-Rel, p105/p50, and p100/p52 that homo- and heterodimerize to regulate transcription of target genes. In the canonical pathway under basal conditions, p65-p50 dimers are bound to IκBα in the cytoplasm. Stimuli such as TNF-α or IL-1 lead to activation of the IKK complex, which consists of two catalytic subunits, IKKα and IKKβ, as well as a scaffolding subunit, IKKγ or NEMO. IKK phosphorylates IκBα, leading to its ubiquitination and proteasomal degradation, which allows NF-κB to accumulate in the nucleus. The non-canonical pathway is activated by stimuli crucial for lymphoid development such as BAFF or CD40. Here, the precursor p100 acts as an IκB molecule bound to RelB. Upon activation, IKKα phosphorylates p100, leading to its cleavage to produce p52. The active RelB-p52 dimer can then regulate transcription of target genes [15, 16]. Originally identified for its role in inflammatory signaling, the NF-κB pathway has since been demonstrated to be activated in various forms of cancer and is thought to contribute to the malignant phenotype through dysregulation of important biological processes such as proliferation, angiogenesis, apoptosis, and cell survival [16–19].

Within the nervous system, NF-κB is typically considered to be inactive, but activated in cases of injury or inflammation, consistent with its canonical function in other tissues [20, 21]. In normal neural stem cells (NSCs), TNFα has been shown to induce proliferation through NF-κB [22]. Kaus et al. [23] have shown that as NSCs acquire the ability to proliferate independent of exogenous growth factors, these cells demonstrate increased NF-κB activity. In GBM, NF-κB has been reported to regulate survival, invasion, and resistance to both radiation and chemotherapy [24–28]. *PTEN* deletion and *EGFR* amplification and/or mutation are two of the most common genetic alterations in GBM and both can lead to increased NF-κB activation [28, 29]. Additionally, TGF-β signaling has been demonstrated to contribute to GSC maintenance through the upregulation of LIF, Sox2, and Sox4 [30, 31]. While the TGF-β and NF-κB pathways are thought to antagonize each other in some settings, there is evidence of their cooperation within tumors, including GBM [32, 33]. Others have identified alterations in the NF-κB pathway itself, with a subset of GBMs harboring monoallelic *NFKBIA* (gene name for IκBα) deletions and others expressing high levels of miR-30e* which targets IκBα [34, 35]. Consistent with the involvement of NF-κB signaling in GBM, recent work demonstrated that treatment with a NEMO-binding domain (NBD) peptide that blocks interactions between NEMO and IKKα/β slowed tumor growth in both a human glioma xenograft and a genetic mouse model of glioma [36].

NF-κB activity has been associated with CSCs in several cancer [37–42], and in GBM the NF-κB targets IL-6 and A20 have been shown to contribute to the maintenance of GSCs [43, 44]. When cells are grown in CSC-permissive conditions instead of monolayers, there is an upregulation of NF-κB activity as seen through p65 phosphorylation and target gene expression [45]. Other data suggest that inducing differentiation of GSCs increases NF-κB activity. However, NF-κB inhibition accelerates differentiation, suggesting a role for this pathway in maintaining the cells in a more stem-like state [46].

In this study, we sought to investigate the role of the NF-κB pathway in GSCs directly. We find that phosphorylation of the p65 (RelA) subunit of NF-κB is elevated in CD133+ GBM cells as compared to CD133- cells. Targeting NF-κB signaling either genetically or pharmacologically impairs self-renewal in primary tumorsphere assays and in limiting dilution assays. Interestingly, both canonical and non-canonical NF-κB pathways contribute to the GSC phenotype. Our results indicate that one source of NF-κB activation in GSCs is a TGF-β signaling pathway acting through TAK1. Using an *ex vivo* brain slice co-culture model, we show that NF-κB contributes to the growth and survival of tumorspheres. Our findings indicate that NF-κB signaling is a key therapeutic target controlling GSCs.

## RESULTS

### NF-κB is preferentially activated in CD133+ cells of GBM explants

**Figure 1:**
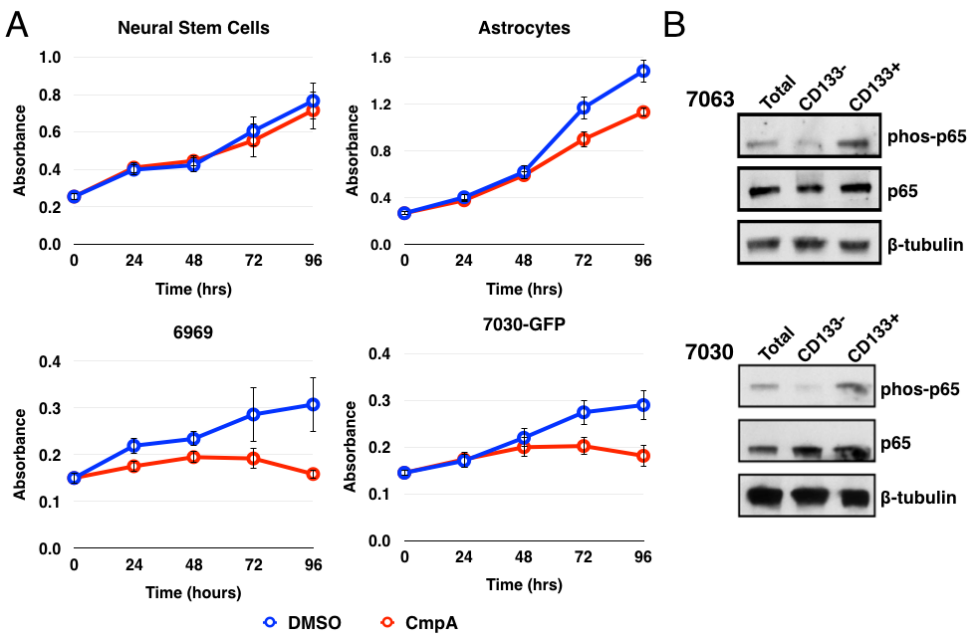
NF-κB is preferentially activated in CD133+ glioblastoma stem cells **A.** MTS assay using normal neural stem cells, astrocytes, or two GBM explants: 6969 and 7030. Cells were treated daily with DMSO or 5 μM Compound A and analyzed every 24 hours for 96 hours. Data are represented as the mean ± s.d. and are representative of three independent experiments. **B.** Analysis of total cells, isolated CD133-, or CD133+ cells by immunoblot for phosphorylation of p65. Quantification of the immunoblots for the ratio of phospho-p65 to total p65 is: 7063 total: 1; CD133-: 0.23; CD133+: 1.77; 7030 total: 1; CD133-: 0.19; CD133+: 1.15.

Given that NF-κB is not highly active in normal brain tissue, we first compared the growth of normal neural stem cells and astrocytes to that of patient-derived GBM explant cultures. We treated cells with DMSO or with the selective IKKβ antagonist Compound A [47] daily for 5 days and measured cell viability by MTS assay (Figure 1A). Results demonstrate that growth was impaired in the GBM explant cultures by NF-κB inhibition but not in the normal neural stem cell or astrocyte cultures, suggesting that NF-κB inhibition preferentially targets tumor cells over normal brain cells. Since GBM was one of the first solid tumors in which CSCs were identified [3, 4] and based on our previous work implicating NF-κB signaling in breast CSCs [39], we next asked if these findings could be explained by a role for NF-κB signaling in the growth of GSCs. To study the stem cell-like *vs*. non-stem cell-like subpopulation of glioblastoma cells, we isolated CD133+ and CD133- fractions from the human glioblastoma explant cultures using magnetic beads. Interestingly, the CD133+ cells exhibited elevated levels of p65 phosphorylation, consistent with increased or altered activity of the canonical NF-κB pathway (Figure 1B).

### Inhibition of NF-κB reduces tumorsphere formation

**Figure 2:**
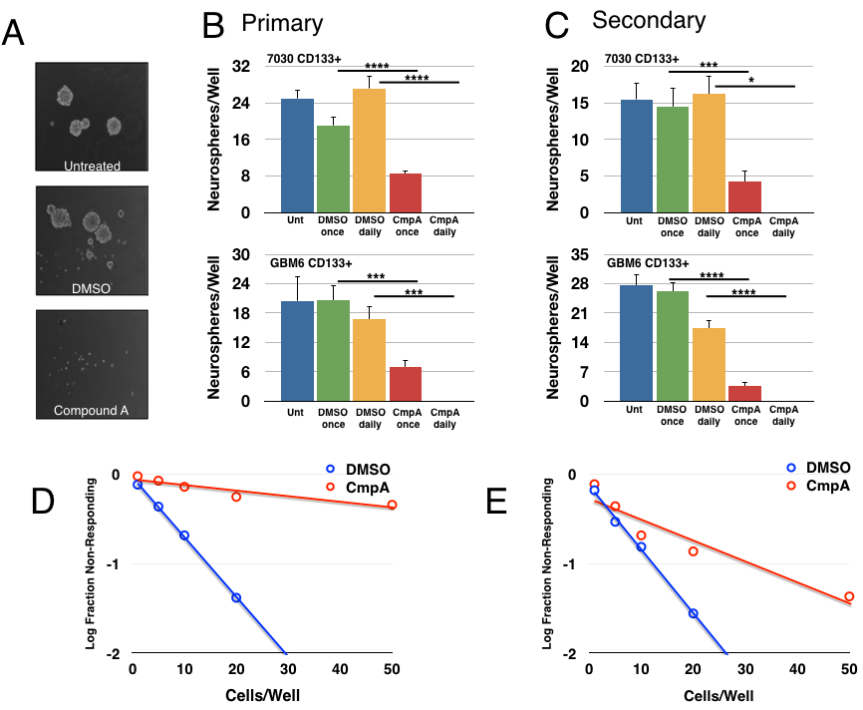
Pharmacological inhibition of the IKK/NF-κB pathway decreases tumorsphere formation 7030 CD133+ cells plated for a tumorsphere assay and treated as indicated (DMSO or 5μM Compound A, once or daily). After one week of growth, tumorsphere formation was analyzed. **A.** Representative images of tumorsphere formation after daily treatment. **B.** Quantification of primary tumorspheres formed per well for two explants. Data are represented as mean ± SEM, ****p < 0.0001, ***p < 0.001, *p < 0.05 by *t*-test. **C.** Primary tumorspheres were dissociated, replated, and treated again as indicated. Secondary tumorsphere formation was quantified after another week of growth. Data are represented as mean ± SEM, ****p < 0.0001, ***p < 0.001, *p < 0.05 by *t*-test. **D.**, **E.** Tumorsphere formation was measured through a limiting dilution assay with 7030 **D.** or GBM6 **E.** CD133+ cells plated at 100, 50, 20, 10, 5, or 1 cell(s)/well and treated with DMSO or 5μM Compound A (7030: *n* = 48 wells/condition; *p* = 2.02×10^−47^; GBM6: n≥116wells/condition; *p* = 2.23×10^−11^).

We next examined whether NF-κB was important in tumorsphere formation, which is an *in vitro* test for stem cell-like activity. After isolation, CD133+ cells were plated at a low density and allowed to form spheroids for one week prior to quantification. Daily treatment with Compound A abrogated tumorsphere formation completely. Importantly, a single treatment with Compound A at the beginning of the experiment was sufficient to reduce tumorsphere formation, suggesting that even transient loss of NF-κB activity could affect stem-like activity (Figure 2A, 2B). In order to further address the effects on self-renewal, primary tumorspheres from the first week of growth were dissociated, re-plated, and then subjected to the same treatments. Treatment with Compound A also reduced secondary tumorsphere formation, again consistent with a role for NF-κB activity in the stem-cell fraction (Figure 2C). Finally, we assayed tumorsphere formation by using a limiting dilution assay with a range of cell concentrations from 1-100 cell(s)/well. For both 7030 and GBM6 CD133+ cells, treatment with Compound A significantly reduced the ability of GBM stem cells to form tumorspheres (Figure 2D, 2E).

**Figure 3:**
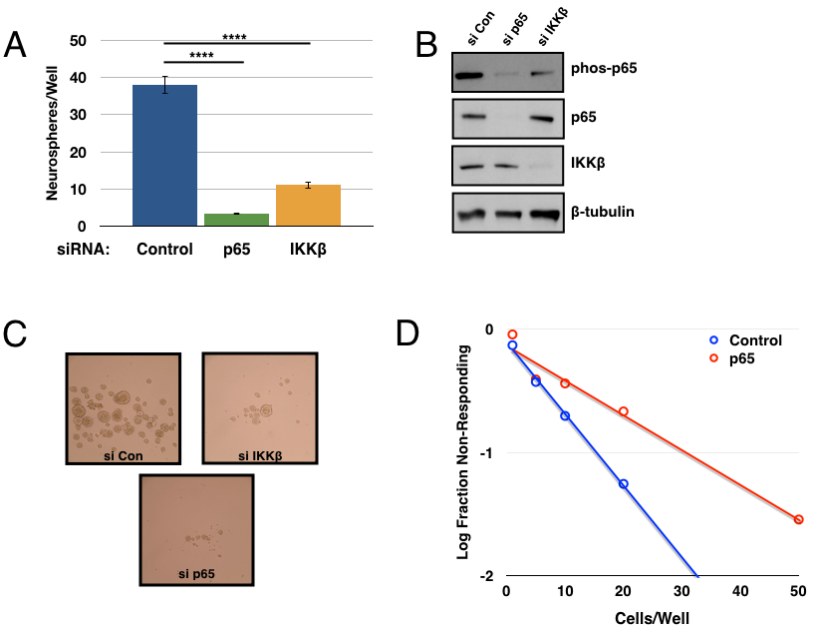
Genetic inhibition of the IKK/NF-κB pathway decreases tumorsphere formation 7030 CD133+ cells were transfected with siRNA (control, p65, or IKKβ) and then plated for tumorsphere assays **A.** Quantification of tumorspheres formed per well after one week of growth. Data are represented as the mean ± SEM, ****p < 0.0001 by t-test. **B.** Transfected cells analyzed by immunoblot for p-p65, p65, IKKβ, and β-tubulin. **C.** Representative images of tumorsphere formation after daily treatment. **D.** Tumorsphere formation was measured through a limiting dilution assay with GBM6 CD133+ cells plated at 100, 50, 20, 10, 5, or 1 cell(s)/well following transfection with control or p65 siRNA (n≥70 wells; *p* = 1.76×10^−6^).

In order to validate the on-target activity of Compound A in inhibiting IKK, we repeated these assays using siRNA-mediated knockdown of IKKβ and/or p65. Knockdown of either protein resulted in a decrease in p65 phosphorylation, as well as a decrease in tumorsphere formation (Figure 3A-3C). The inhibitory effects of p65 knockdown were confirmed in the limiting dilution assay consistent with previous results (Figure 3D). Taken together, these results strongly implicate a role for the NF-κB pathway in GSC propagation and self-renewal.

### Multiple NF-κB subunits contribute to GSC maintenance

**Figure 4:**
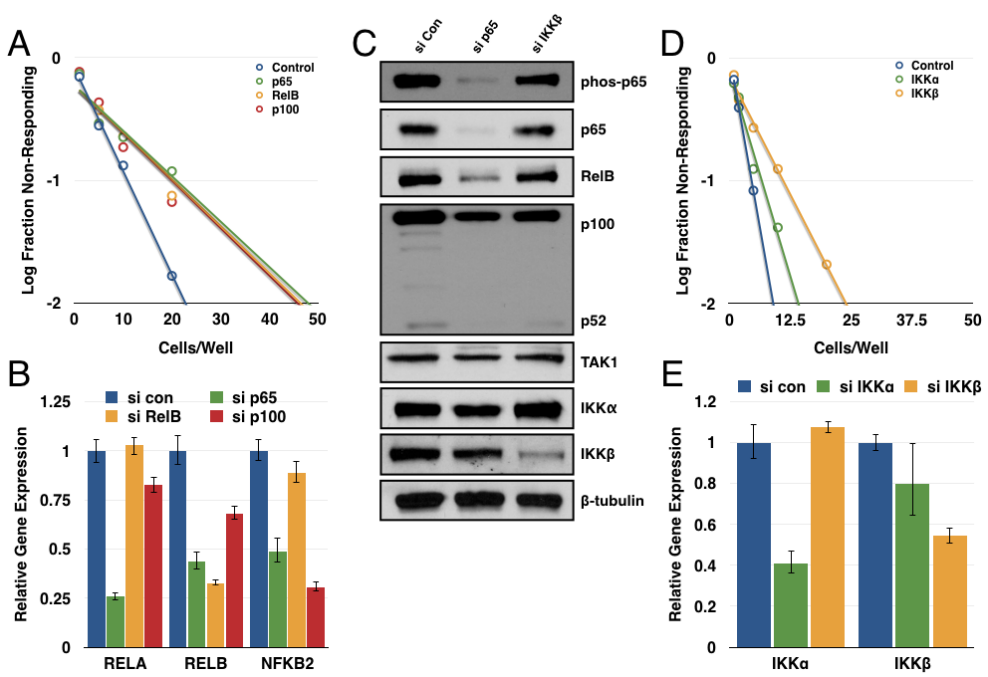
Multiple NF-κB subunits contribute to tumorsphere formation **A.** GBM6 CD133+ cells were transfected with siRNA control or targeting p65, RelB, or p100. Subsequently, cells were plated out for limiting dilution assay at 100, 50, 20, 10, 5, or 1 cell(s)/well and scored for the presence or absence of tumorspheres following one week of growth (n≥95 wells/condition; p65 *vs*. control *p* = 2.26×10^−5^; RelB *vs*. control *p* = 6.44x10^−5^; p100 *vs*. control *p* = 3.82x10^−5^). **B.** Quantitative real-time PCR was performed to analyze expression of *RELA, RELB, or NFKB2* in siRNA-transfected cells normalized to *GUSB* expression. **C.** Expression of canonical and non-canonical NF-κB components was analyzed by immunoblot in 7030 CD133+ cells transfected with siRNA (control, p65, or IKKβ). **D.** GBM6 CD133+ cells were transfected with siRNA (control, IKKα, or IKKβ). Subsequently, cells were plated out for limiting dilution assay at 50, 20, 10, 5, 2, or 1 cell(s)/well and scored for the presence or absence of tumorspheres following one week of growth (n≥48 wells/condition; IKKα *vs*. control *p* = 0.192; IKKβ *vs*. control *p* = 6.01x10^−5^). **E.** Quantitative real-time PCR was performed to analyze expression of *CHUK and IKBKB* in siRNA-transfected cells normalized to *GUSB* expression.

Up to this point, our studies have focused on the more highly studied canonical NF-κB pathway. We next sought to determine if the non-canonical NF-κB pathway, driven by a RelB-p52 dimer, could be contributing to GSC maintenance as well. Accordingly, GBM6 CD133+ cells were transfected with siRNAs targeting p65, p100/p52, or RelB. Each of these subunits produced a substantial decrease in tumorsphere formation in a limiting dilution assay (Figure 4A). Knockdown efficiency was confirmed through qPCR (Figure 4B). These results suggest that both the canonical and non-canonical NF-κB pathways contribute to GSC maintenance.

Consistent with the literature, knockdown of p65 resulted in decreased transcription of both RelB and p100 [48, 49]. We found that knockdown of p65 and IKKβ decreased expression of the non-canonical subunits at the protein level as well (Figure 4C). Interestingly, knockdown of IKKβ but not IKKα significantly decreased tumorsphere formation in a limiting dilution assay (Figure 4D, 4E). Under normal culture conditions, no NIK expression was detected even with the addition of the proteasome inhibitor MG132. Perhaps not surprisingly, we did not see consistent results when attempting to knockdown NIK and assay tumorsphere formation (data not shown). It is possible that the residual IKKα following siRNA knockdown is sufficient for p100 processing to p52 and that a more complete knockout would reveal a larger effect for IKKα. Alternatively, p100 could be functioning in a separate role rather than simply being the precursor to p52, which is what is being observed in these experiments. These possibilities are further explored in the discussion. Nonetheless, these results suggest that transcriptional regulation of the non-canonical subunits (RelB and p100) by the canonical subunit (p65) could be a significant aspect of non-canonical pathway regulation in GSCs.

### TAK1 activates the NF-κB pathway to promote GSC function

**Figure 5:**
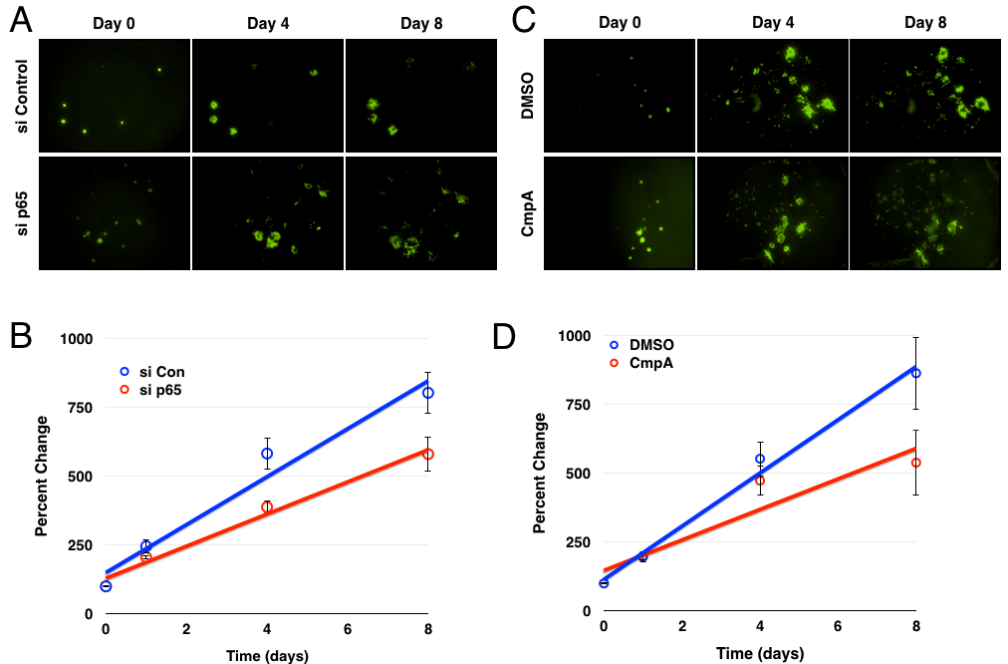
TAK1 activates the NF-κB pathway to promote glioblastoma stem cell function **A.** 6969 or 7030 cells were transfected with 3x-κB luciferase reporter, treated with the indicated inhibitors for 24 hours, then harvest and analyzed for luciferase activity (*n* = 3; **p < 0.0001 by *t*-test, error bars represent SEM) **B.** Quantification of tumorsphere formation in GBM6 CD133+ cells following treatment with either (5Z)-7-oxozeaenol or NG25 either once or daily. Data are represented as the mean ± SEM, **p < 0.0001 by *t*-test. **C.** Limiting dilution assay with GBM6 CD133+ cells transfected with siRNA control or TAK1 (*n* = 72 wells/condition; *p* < 0.05). **D.** Limiting dilution assay following treatment of GBM6 CD133+ cells with structurally distinct TAK1 inhibitors: 2.5 μM (5Z)-7-oxozeaenol or 2 μM NG-25 (n≥90wells/condition; 5Z *vs*. DMSO *p* = 1.01×10^−19^, NG *vs*. DMSO *p* = 5.29×10^−10^).

Next, we examined an upstream activator of the NF-κB pathway, transforming growth factor-β-activated kinase 1 (TAK1), which is known to activate the IKK complex following cytokine stimulation as well as in some oncogenic settings [50–54]. We first showed that use of two structurally distinct TAK1 inhibitors ((5Z)-7-oxozeanol and NG-25) decreased the expression of an NF-κB luciferase reporter in both GBM explant cultures (Figure 5A). In tumorsphere formation assays, treatment with either (5Z)-7-oxozeanol or NG-25 decreased tumorsphere formation in GBM 7030 CD133+ cells with a single treatment and to an even greater extent with daily treatment (Figure 5B). We then extended these studies in limiting dilution assays, finding that both siRNA against TAK1 and the TAK1 inhibitors resulted in significant decreases in tumorsphere formation (Figure 5C, 5D).

### TGF-β is one source of NF-κB activation

**Figure 6:**
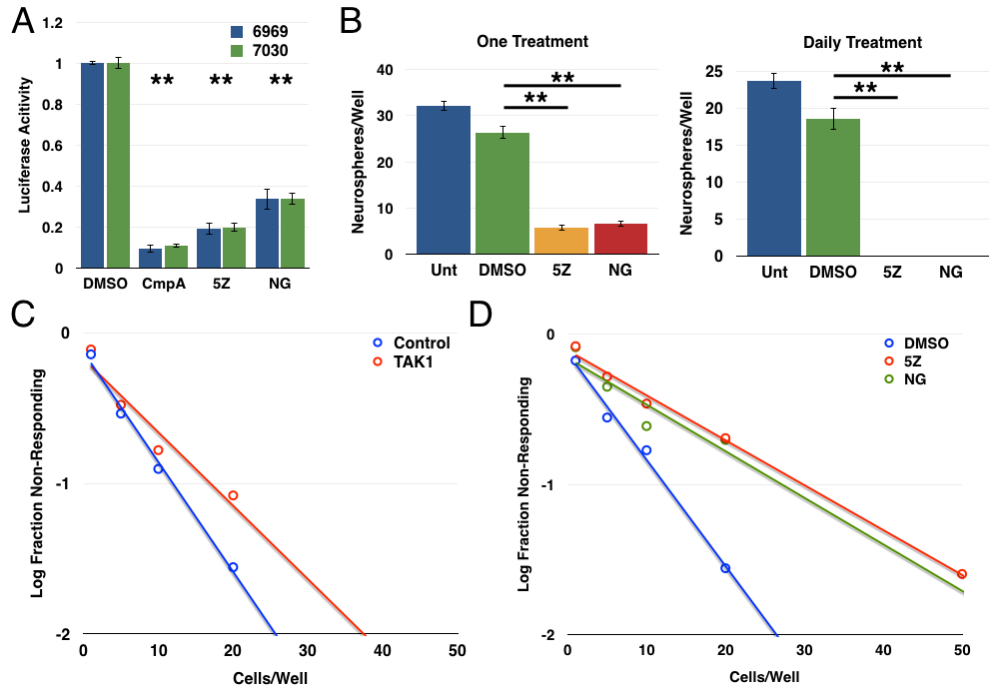
TGF-β is one source of NF-κB activation in GBM **A.** 6969 and GBM6 explants were stimulated with 10ng/mL TGF-β for 6 or 24 hours, then analyzed by immunoblotting for phosphorylation of Smad and p65. **B.** 6969 and GBM6 explants were treated with 10 μM SB431542, a TGF-βR1 inhibitor, for 6 or 24 hours, then analyzed by immunoblotting for p65 phosphorylation. **C.** 6969 or 7030 cells were transfected with 3x-κB luciferase reporter and treated with DMSO or 10 μM SB431542 for 24 hours, then harvested and analyzed for luciferase activity (*n* = 3, **p < 0.0001, *p < 0.01 by *t*-test, error bars represent SEM). **D.** Quantification of tumorsphere formation in GBM6 CD133+ cells following treatment with 10 μM SB431542 either once or daily. Data are represented as the mean ± SEM, **p < 0.0001 by *t*-test.

As the cognate activator of TAK1 is transforming growth factor-β (TGF-β) itself, and as the TGF-β pathway has previously been implicated in regulating GBM CSCs [30, 31], we next investigated a potential link between TGF-β and NF-κB in the setting of GBM. Studies showed that treatment with exogenous TGF-β led to an increase in Smad phosphorylation (as expected) and in p65 phosphorylation in GBM explant cultures (Figure 6A). Conversely, addition of a TGF-βR1 inhibitor (SB431542) led to a decrease in p65 phosphorylation (Figure 6B). Additionally, the TGF-βR1 inhibitor induced a small but consistent decrease in luciferase activity from an NF-κB reporter in GBM explant cultures (Figure 6C). Together, these results suggest an autocrine/paracrine role for TGF-β in maintaining NF-κB signaling in these GBM explant cultures. Use of the TGF-βR1 inhibitor in a tumorsphere assay leads to a significant decrease in tumorsphere formation in GBM6 CD133+ cells (Figure 6D). These results suggest that TGF-β can activate NF-κB signaling in these GBM explants. However, given the scale of these changes, it is likely only one of several sources of NF-κB activation, consistent with the pleiotropic nature of this pathway. Similarly, given the more drastic effects seen in the tumorsphere assay with the TGF-βR1 inhibitor, it is likely that the TGF-β pathway is also mediating additional factors involved in GSC biology.

### Inhibition of the IKK/NF-κB pathway decreases glioblastoma growth *ex vivo*

**Figure 7:**
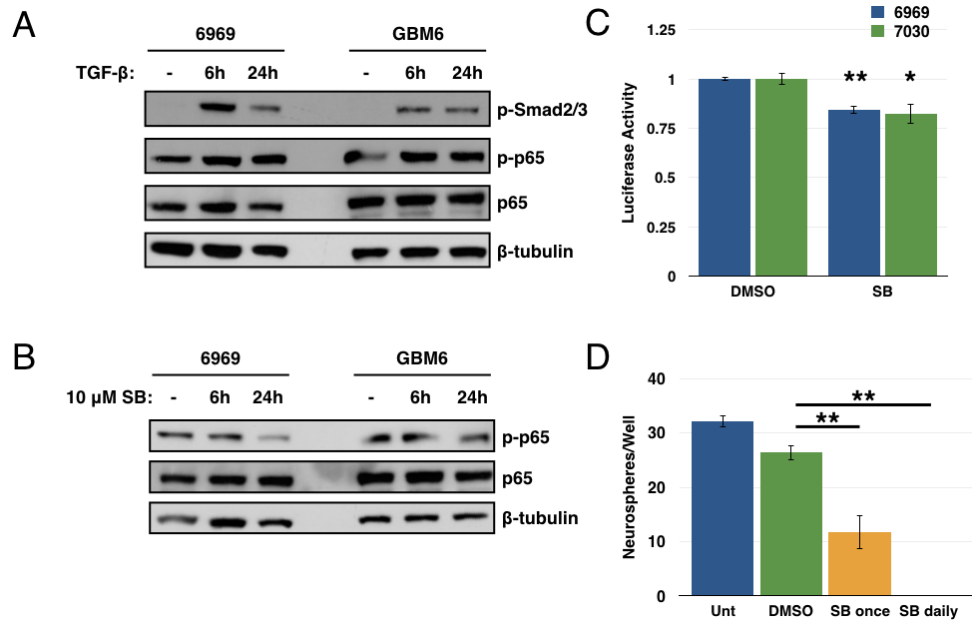
Inhibition of the IKK/NF-κB pathway decreases glioblastoma growth/survival *ex vivo.* **A.** Images of GFP+ GBM6 cells following siRNA transfection and brain slice culture. **B.** Average percent change in GFP+ area over the course of the experiment (*n* = 14 for control, 13 for p65; *p* < 0.005 by linear regression; error bars represent SEM) **C.** Images of GFP+ GBM6 cells over the course of eight days of culture on brain slices following treatment with DMSO or Compound A. **D.** Average percent change in GFP+ area over the course of the experiment (*n* = 12; *p* < 0.01 by linear regression; error bars represent SEM)

Finally, we sought to validate the *in vitro* studies in a more biologically relevant setting, turning to an *ex vivo* organotypic brain slice preparation which has been used previously [55, 56]. This methodology provided the opportunity for higher throughput analysis, as well as a level of longitudinal imaging not typically available *in vivo.* For these experiments, neonatal rat brain tissues were sectioned into 250 μm coronal slices, then plated on top of an agar medium as previously described [57]. GBM6-GFP spheres were then engrafted onto the upper surfaces of these these brain slice explants shortly after slicing. A first round of imaging was then completed within a few hours of plating to establish a baseline for GBM tumor growth these brain slices. The brain slices were then imaged daily for up to eight days and the GFP-positive areas were quantified for each slice and on each day using ImageJ software. Representative images show that implanted GBM tumorspheres progressively grow and invade the surrounding brain tissues over the course of the experiment (Figure 7A).

To examine a role for NF-κB in GBM growth within these *ex vivo* orthotopic xenografts, GBM6-GFP cells were transfected with either control or p65 siRNA twenty-four hours prior to tumorsphere formation and brain slice implantation. Quantification of tumorsphere cross-sectional areas indicated that transfection with p65 siRNA significantly inhibited the growth rate of the implanted GBM tumorspheres over the course of the experiment (Figure 7B). Similarly, addition of the IKKβ inhibitor to the implanted brain slice cultures also led to significant inhibition of GBM tumorsphere growth (Figure 7C, 7D).

## DISCUSSION

As with many other tumor types, glioblastoma is characterized by a hierarchical organization of cells, including a subpopulation of so-called cancer stem cells (or tumor-initiating cells). These cells promote tumor initiation and recurrence, drive invasion and metastasis, and demonstrate increased resistance to radiation and chemotherapy [3, 4, 11–14]. It is crucial to investigate the signaling pathways responsible for these phenotypic differences from the bulk of the tumor, both to establish insight into mechanisms that promote these cells and to potentially identify therapeutic targets for disease control. In this study, we demonstrate the involvement of the IKK/NF-κB pathway in the function of GSCs. We identified a TGF-β/TAK1 axis as one source of NF-κB activation in these cells. TAK1 is a well-established activator of IKK and there is a precedent for cooperation between the TGF-β and NF-κB pathways in GBM [32, 33]. Nonetheless, it is likely that other sources for NF-κB activation exist, such as cytokines, which utilize TAK1 as a mediator of NF-κB activation. Indeed, common genetic alterations seen in GBM (*PTEN* deletion, *EGFR* amplification and mutation, and monoallelic *NFKBIA* deletion) have all been connected to enhanced NF-κB activation [28, 34, 58–63]. Our data from the *ex vivo* co-culture experiments show greater effects following IKK inhibition in the whole slice rather than just the cancer cells. Given the extensive number of cytokines and chemokines regulated by NF-κB, it is very likely that this pathway impacts the interactions between the tumor, microglia, and infiltrating immune cells, potentially related to the finding that GSCs promote tumor evasion *via* immunosuppression [64, 65]. Consistent with our studies overall, Verma and colleagues showed that treatment with an NBD peptide impaired tumor growth in both a human glioma xenograft and a genetic mouse model of glioma [36], focusing on inhibition of canonical NF-κB signaling through disruption of the IKK complex. Thus, the effects of IKK inhibition in GBM models may function, at least partly, at the level of GSCs.

As a family of transcription factors, the NF-κB pathway is likely mediating a variety of downstream effects in GBM cells. We found that both the canonical and non-canonical branches contribute to GSC biology. Our results show larger effects of targeting RelB and p100 compared to IKKα, as well as decreased expression of the non-canonical subunits following knockdown of p65. One possibility is that since siRNA knockdown is incomplete, the remaining IKKα is still capable of driving sufficient processing of p100 into p52 and that use of a CRISPR knockout would produce a more substantial phenotype. Alternatively, since p52 is generated from p100 protein, knockdown at the gene level affects both p100 and p52 levels. Thus, we could actually be observing an independent effect of loss of p100 that is not dependent on IKKα for processing into the active p52 subunit. For example, a recent report demonstrated that p100 can interact with ERK2 and inhibit its nuclear localization [66], providing a precedent for p100-specific activities.

At this point, it is still unknown whether these two pathways are acting on common or separate targets. The dimerization and DNA binding patterns of NF-κB subunits remain complex areas of study [67–72]. The non-canonical pathway has been shown to have distinct functions in oncogenesis, such as driving growth and invasion of mesenchymal glioma and regulation of the mutant C250T *TERT* promoter [73, 74]. Antagonism of p53 through NF-κB regulation of Mdm2 expression has been well-established and can contribute to chemotherapy resistance [75–77]. Additionally, NF-κB is known to regulate target genes related to several GSC functions including survival (*BCL2*, *BCL2L1*), invasion (*IL6, IL8, CCL2, MMP2/3/9*), and resistance to therapy (*MGMT, TNFAIP3, TRADD*) [78–85]. Gene expression analysis showed NF-κB regulation of some of these targets, however neither the expression nor the NF-κB-dependency was limited to the CD133+ population of cells. It is likely that the effects on GSCs observed following NF-κB inhibition result from a combination of these and other genes. Given its central position in GSC signaling, the IKK/NF-κB pathway is proposed as a target for therapeutic intervention in glioblastoma.

## MATERIALS AND METHODS

### Cell culture, CD133+ isolation, and reagents

The human glioblastoma explants (6969, 7030, 7063, and GBM6) were obtained from UCSF and maintained in Neurobasal medium (Invitrogen), supplemented with B27 without Vitamin A, L-glutamine, 20ng/mL EGF, 40ng/mL FGF, and penicillin/streptomycin. To dissociate tumorspheres, cells were incubated with Accutase (Sigma) in a 37°C water bath for 10 minutes, then plated as desired. To isolate CD133+ cells, cells were dissociated with Accutase and passed over a pre-separation filter to achieve single cell suspension. Dead cells were removed using the Dead Cell Removal Kit according to manufacturer's instructions (Miltenyi). Remaining cells were incubated with CD133 magnetic microbeads (Miltenyi) for 30 minutes, resuspended in MACS buffer and passed over two LS columns consecutively. Non-retained cells were saved for the CD133- fraction. After washing, the column was removed from the magnet and retained cells were expelled from the column, counted, and plated for experiments. Normal human astrocytes were a kind gift from Dr. Russell Pieper [86, 87] and were maintained in DMEM media with 10% FBS and 1% penicillin/streptomycin. Neural stem cells (Millipore) were maintained in RenCell NSC maintenance media and grown on laminin-coated plates.

### 3-(4,5-Dimethylthiazol-2-yl)-5-(3-carboxymethoxyphenyl)-2-(4-sulfophenyl)-2H-tetrazolium cellular proliferation assay

Cells were seeded at 2000 or 3000 cells per well in 96-well plates, then treated with DMSO or Compound A daily as indicated. At each time point, 3-(4,5-dimethylthiazol-2-yl)-5-(3-carboxymethoxy-phenyl)-2-(4-sulfophenyl)-2H-tetrazolium (MTS) compound (Promega) was added and absorbance was read at 490 nm on a Versamax Microplate Reader (Molecular Devices).

### Luciferase assay

Cells were transfected with 3x-κB luciferase reporter plasmid [88] using FuGENE HD (Promega). Six hours post-transfection, cells were separated into 12-well plates and treated in duplicate with inhibitors as indicated for 24 hours. Cells were lysed in 150 μL of Passive Lysis Buffer, then 20 μL of lysate was used for analysis in triplicate using the Luciferase Assay System (Promega). Luciferase signal was read on a Synergy2 plate reader (Biotek), and then normalized to protein content of each well based on a Bradford assay.

### Western blotting

Whole cell extracts were prepared by collecting cells, washing with cold PBS, then suspending in cold lysis buffer (1% NP-40, 20 mM Tris, 138 mM NaCl, 2mM EDTA, 10% glycerol) on ice for 10 minutes, followed by 10 minutes of centrifugation to remove insoluble components. Protein was quantitated by Bradford assay (Biorad). Equal amounts of lysate (25-50 μg) were separated by SDS-PAGE, transferred to nitrocellulose membranes, and blocked for 1 hour in 5% milk. Membranes were incubated with primary antibody overnight at 4°C, then incubated with secondary antibody for 1 hour at room temperature and developed using ECL reagent (GE). Antibodies used were phospho-Smad2/3 (S465, 467/S423, 425), phospho-p65 (S536), p65, IKKβ, RelB (Cell Signaling Technology), IKKα, p100/p52 (Millipore), TAK1 and β-tubulin (Santa Cruz Biotechnology).

### siRNA transfection

Human siRNA targeting *RELA* (M-003533-02), *RELB* (M-004767-02), *NFKB2* (M-003918-02), *CHUK* (M-003473-02), *IKBKB* (M-003503-03), *MAP3K7* (M-003790-06), or control #3 (D001201-03) was purchased from Thermo/Dharmafect. DharmaFECT reagent 1 was used to transfect siRNA into cells according to the manufacturer's instructions. Six hours post-transfection, the media was changed on the cells. Cells were harvested 48-72 hours post-transfection for analysis by quantitative real-time PCR or Western blot.

### Quantitative real-time PCR

RNA extracts were obtained from cells using the RNeasy Plus Kit (Qiagen). Two micrograms of RNA were reverse transcribed using random primers and MMLV reverse transcriptase (Invitrogen). Real-time PCR was performed using Taqman Gene Expression Assay primer-probe sets for *GUSB* (Hs99999908_m1), *RELA* (Hs00153294_m1), *RELB* (Hs00232399_m1), *NFKB2* (Hs01028901_g1), *CHUK* (Hs00989502_m1), and *IKBKB* (Hs00233287_m1) and relative quantification was determined using the ΔΔC_t_ method.

### Tumorsphere and limiting dilution assays

For tumorsphere assays, cells were dissociated into single cell suspension using Accutase. Cells were counted and plated at 100 cells per well in 24-well tissue culture plates. Cells were treated with inhibitors as indicated (once or daily for the duration of the experiment): 5 μM Compound A, 2.5 μM (5Z)-7-oxozeanol (Tocris), 2 μM NG25 (MedChem Express), and 10 μM SB431542 (Tocris). Following one week of growth, tumorspheres were viewed on the microscope and quantified. For limiting dilution assays, cells were serially diluted to be plated at 100, 50, 20, 10, 5, 2 or 1 cell(s)/well in 96-well plates. After one week of growth, wells were scored for the presence or absence of spheres. Extreme limiting dilution analysis was performed as previously described [89], using software available at http://bioinf.wehi.edu.au/software/elda/.

### Brain slice explantation and tumorsphere implantation

Coronal brain slices (250 μm thick) from postnatal day 10 Sprague-Dawley rat pups of either sex (Charles River, Wilmington, MA) were prepared and explanted in organotypic culture as previously described [57]. Animals were sacrificed in accordance with NIH guidelines and under Duke IACUC approval and oversight. Briefly, brain tissues were sliced in ice-cold artificial cerebrospinal fluid and plated in interface configuration atop culture medium (Neurobasal A medium supplemented with 15% heat-inactivated horse serum, 10 mM KCl, 10 mM HEPES, 100 U/ml penicillin/streptomycin, 1 mM sodium pyruvate, and 1 mM L-glutamine) semi-solidified in 0.5% reagent-grade agarose in 12-well plates. Brain slice explants were incubated under 5% CO_2_ at 37 °C for up to 8 days as indicated. Compound A or DMSO-only vehicle was added to culture medium at the time of brain slice explantation. Small groups of GBM tumorspheres were implanted shortly thereafter by direct application to the upper surfaces of each brain slice. An epifluorescence stereomicroscope was used to obtain images through the course of experiments. ImageJ was used to quantify the area of GFP+ cells.
